# Approach to SARS-CoV-2 Vaccination in Patients With Multiple Sclerosis

**DOI:** 10.3389/fimmu.2021.701752

**Published:** 2021-06-21

**Authors:** Christina Woopen, Katharina Schleußner, Katja Akgün, Tjalf Ziemssen

**Affiliations:** Center of Clinical Neuroscience, Department of Neurology, University Hospital Carl Gustav Carus Dresden, Technical University of Dresden, Dresden, Germany

**Keywords:** severe acute respiratory syndrome coronavirus 2 (SARS-CoV-2), coronavirus disease (COVID-19), vaccination, multiple sclerosis, immunotherapy, immunomodulation, CD20, immune response

## Abstract

For more than a year now, severe acute respiratory syndrome coronavirus 2 (SARS-CoV-2) has been causing the coronavirus disease (COVID-19) pandemic with high mortality and detrimental effects on society, economy, and individual lives. Great hopes are being placed on vaccination as one of the most potent escape strategies from the pandemic and multiple vaccines are already in clinical use. However, there is still a lot of insecurity about the safety and efficacy of vaccines in patients with autoimmune diseases like multiple sclerosis (MS), especially under treatment with immunomodulatory or immunosuppressive drugs. We propose strategic approaches to SARS-CoV-2 vaccination management in MS patients and encourage fellow physicians to measure the immune response in their patients. Notably, both humoral and cellular responses should be considered since the immunological equivalent for protection from SARS-CoV-2 after infection or vaccination still remains undefined and will most likely involve antiviral cellular immunity. It is important to gain insights into the vaccine response of immunocompromised patients in order to be able to deduce sensible strategies for vaccination in the future.

## Introduction

Severe acute respiratory syndrome coronavirus 2 (SARS-CoV-2) is the viral pathogen that causes coronavirus disease 2019 (COVID-19). Typical clinical manifestations range from mild upper respiratory tract symptoms to respiratory failure, severe COVID-19 disease can even lead to septic shock and multi-organ failure. Neurological complications encompass cerebrovascular disease, encephalopathy, seizures, and Guillain-Barré syndrome, amongst others (1, 2). Since the emergence of SARS-CoV-2 in Wuhan, China, in December 2019, the virus has spread worldwide and led to a persistent pandemic with more than 2.960.000 deaths and an enormous social and economic burden (3, 4). The protective capacity of the adaptive immune response to SARS-CoV-2 seems to depend not only on virus-specific antibodies but also on the cellular response to infection (5–9) and vaccination (10–12).

The rapid development of several vaccines against SARS-CoV-2 is a major achievement of the scientific community (13). As of April 2021, three vaccines have been approved for emergency use by the United States Food and Drug Administration: the messenger ribonucleic acid (mRNA) vaccines BNT162b2 developed by Pfizer-BioNTech and mRNA-1273 by Moderna, and the vector vaccine Ad26.COV2.S by Janssen (14). Additionally to the three aforementioned vaccines, the European Commission granted conditional marketing authorization for a fourth vaccine also based on viral vector technology: ChAdOx1 nCoV-19 (AZD1222) by Oxford-AstraZeneca (15). Vaccines based on DNA, viral protein, and inactivated virus platforms are being administered in other parts of the world (16). Another 88 vaccines are currently in clinical and 184 in pre-clinical development worldwide (17).

Multiple sclerosis (MS) is an autoimmune-mediated disorder defined by a timely and locally disseminated inflammation of the central nervous system (18). 2.8 million people worldwide are affected by MS which is moreover the most common neurological cause of disability in young adults (19). In most cases, the disease is initially characterized by relapses but it often evolves into a chronic progressive course over time. For around 15% of patients, the disease takes a progressive clinical course from the beginning (20). Fortunately, several different types of immunomodulatory and immunosuppressive therapies have become available over the last decades. Application of these disease-modifying therapies (DMT) significantly reduces relapse frequency but some DMT are also associated with a higher susceptibility to infections (21–23). While MS patients do not have an increased risk of SARS-CoV-2 infection or severe COVID-19 disease per se, the risk is elevated in the presence of comorbidities, higher age, greater MS-associated disability, progressive MS disease course, and under treatment with some types of DMT (24–28). Based on data available up until now, CD20+ B cell-depleting therapies increase the probability of SARS-CoV-2 infection and of severe COVID-19 in MS patients and in patients with rheumatic and musculoskeletal disease (24, 29–31). A trend for an increased risk of contracting COVID-19 symptoms was also reported for patients treated with alemtuzumab and cladribine (32). MS patients with a history of methylprednisolone therapy in the last four weeks had an elevated risk for a worse outcome of COVID-19 disease (29). Patients treated with beta-interferons and glatiramer acetate, in contrast, were shown to have a reduced COVID-19 risk (24). Notably, immunocompromised patients may be confronted with the hazard of viral escape mutations during chronic SARS-CoV-2 infection (33). Infections constitute a major risk for relapse in MS patients (34) and, on top of this, infection-associated relapses are more likely to induce sustained disability compared to relapses not associated with infection (35). Taking this into account, the SARS-CoV-2 pandemic poses an additional threat to MS patients apart from the risks associated with COVID-19 disease itself. SARS-CoV-2 vaccination has recently become available as an effective tool to reduce the risk of infection. However, there is a lot of insecurity about the safety and efficacy of SARS-CoV-2 vaccination among MS patients and practitioners.

## Non-Live Vaccines Are Safe in MS Patients With and Without DMT

Vaccination has been relieving the world of infections with multiple pathogens for more than a century (36). While vaccines have been safely and efficaciously administered for a long time, doubts about the safety of vaccine application in patients with autoimmune disorders have repeatedly arisen and led to insufficient vaccination of MS patients. Case reports suggested an elevated relapse frequency in MS patients after vaccination (37, 38). However, many studies have since been able to show that the employment of non-live vaccines in patients with MS is safe and does not constitute an increased risk for relapse (39–42). This is why current patient care guidelines, e.g. by the American Academy of Neurology (43) or the German Vaccination Commission (44), recommend the application of standard local vaccination schemes also in patients with MS. Likewise, established DMT does not constitute a contraindication to immunization with non-live vaccines.

Vaccines against SARS-CoV-2 currently authorized in Europe and the United States are based on mRNA and viral vector technology and hence belong to the group of non-live vaccines. As they are the first of their type in clinical use, representative data on their application in MS patients are still limited. However, deducing from studies on the safety of other non-live vaccines in patients with MS and from the first reported data on the application of BNT162b2 in MS patients (45), it is very likely that the authorized SARS-CoV-2 vaccines as well as DNA, viral protein, and inactivated virus vaccines will prove to be safe as well (16). Only live attenuated vaccines should be avoided in MS patients under DMT due to a risk of infection. Based on a thorough benefit-risk assessment including first experiences with BNT162b2 and ChAdOx1 nCoV-19 in MS patients in Israel and England, the MS International Federation (46) and the German MS Association (47) recommend vaccinating all eligible MS patients with non-live SARS-CoV-2 vaccines as it is of utmost importance to minimize the infection and concomitant relapse risk in this group.

## Vaccination Is Protective, yet Partly Less Effective Under Certain DMT

Besides the discussed safety issues concerning SARS-CoV-2 immunization in MS patients, the question of vaccine efficacy needs to be addressed. Multiple studies evaluating the effectiveness of different vaccines in MS patients with and without DMT have been conducted and several reviews on this topic are available (30, 48–50):

- For MS patients without DMT, no difference was found in the virus-specific humoral and cellular response to the seasonal influenza vaccine as compared to healthy controls (49, 51–53).
- Beta-interferons are cytokines that unfold their immunomodulatory effects *via* different cascades downstream of the interferon receptor. For treatment with these substances, several studies were able to show that the humoral response to multiple vaccine types is not adversely affected (42, 53–58).
- Glatiramer acetate is a synthetic copolymer consisting of four amino acids that simulates myelin basic protein. Treatment causes immunomodulatory effects on both innate and adaptive immune responses including a shift from a T helper type 1 to a T helper type 2 and regulatory T cell profile. Since pro-inflammatory T helper type 1 responses are usually needed for protective immunity against infectious agents, glatiramer acetate might be expected to impair vaccine responses. However, sufficient immune responses to inactivated seasonal influenza vaccine were mounted in three studies in glatiramer acetate treated MS patients, with a lower protection rate compared to healthy controls in only one of the studies (42, 53, 57).
- Teriflunomide inhibits the enzyme dihydroorotate dehydrogenase which leads to a reduced cell proliferation, especially of activated lymphocytes. For teriflunomide, most or all subjects reached seroprotection in response to different vaccine types (55, 59). In one of the studies, the geometric mean antibody titer after immunization with inactivated rabies vaccine was significantly lower in subjects receiving teriflunomide as compared to the ones receiving placebo, but all reached seroprotective levels so that a clinical relevance of this observation may be doubted (59).
- Dimethyl fumarate reduces inflammation *via* nuclear factor erythroid 2-related factor (Nrf2)-dependent and –independent mechanisms. Regarding dimethyl fumarate, responses to multiple vaccine types were not significantly affected compared to patients taking beta-interferons but only one published study is available for this agent (58).
- Sphingosine-1-phosphate (S1P) receptor modulators prevent the exit of lymphocytes from lymph nodes. Fingolimod and siponimod caused a reduced humoral and cellular immune response to different vaccines according to evidence from several studies (42, 53, 60–62). Based on these findings, it is recommended in the summary of product characteristics of siponimod to discontinue treatment one week before until four weeks after a planned vaccination. As the treatment interruption may provoke MS disease activity, this decision must be based on a thorough benefit-risk-assessment and further data are needed to evaluate efficacy and risk of this strategy. For the newer S1P receptor modulator ozanimod, no studies on vaccination are published yet (48).
- Natalizumab blocks the alpha-4 chain of the very late antigen 4 (VLA-4) on different inflammatory cells hindering them from entering the central nervous system. In natalizumab treatment, two studies did not show significant differences concerning vaccine-specific antibody responses to several types of immunization. Three other studies, however, found lower protection rates after influenza vaccination for natalizumab-treated patients compared to healthy controls (42, 53, 57, 63, 64).
- Alemtuzumab is a monoclonal anti-CD52 antibody leading to depletion of mature lymphocytes. With regard to vaccination in alemtuzumab-treated patients, only one study is available. Here, responses to multiple vaccine types were maintained, but the responding proportion was lower when immunization took place within 6 months of treatment (65).
- Cladribine is a purine analog which causes lymphocyte depletion by interfering with DNA synthesis, repair and cell metabolism. For cladribine, no conclusive data on vaccination are available yet, but multiple studies are currently being conducted (48, 66).
- Ocrelizumab, rituximab, and ofatumumab are anti-CD20 monoclonal antibodies leading to depletion of B cells in the circulation. Concerning treatment with humanized anti-CD20 antibody ocrelizumab, humoral responses to multiple vaccines were attenuated as compared to MS patients with beta-interferon or without treatment. However, immunization was able to elicit seroprotection or a considerable increase in antigen-specific antibody levels in ocrelizumab-treated patients when administered with an interval of 12 weeks to the time point of treatment initiation (67). As for chimeric mouse-human anti-CD20 monoclonal antibody rituximab, similar results with attenuated humoral responses to tetanus toxoid and influenza immunization were reported in patients with rheumatological and hematological disorders. The magnitude of immunization-elicited antibody responses was partly associated with the degree of B cell repletion at the time of vaccine administration (68–70). First data on the humoral response to SARS-CoV-2 mRNA vaccines in patients with chronic inflammatory diseases show reduced or even undetectable virus-specific IgG and neutralizing antibody titers in patients under B cell-depleting therapies (71). Recently, a case was reported in which SARS-CoV-2 mRNA vaccine BNT162b2 failed to prevent COVID-19 in a patient with relapsing MS under ocrelizumab treatment. The patient had received the first vaccine dose approximately two weeks after the last infusion of ocrelizumab, followed by the second vaccine dose around three weeks after the first one. Two and a half weeks after the completed immunization, the patient developed symptoms of a respiratory tract infection and SARS-CoV-2 was detected in a nasopharyngeal swab (72). Overall, the available data suggest that the timing of vaccination in relation to the treatment cycle of B cell depletion may be decisive for the development of a protective immune response. In order to enhance vaccine efficacy, Baker et al. suggest the possibility of stretching the interval between doses of CD20-depleting therapies so that immunization takes place at a time point where immature B cells are already recovering whereas memory B cells are still depleted, the latter preventing re-activity of autoimmune disease (73). In this regard, monthly administered subcutaneous human B cell-depleting agent ofatumumab may offer the advantage of a shorter time span until CD19+ B cell repletion takes place (74). For ocrelizumab treatment, it is often suggested to vaccinate patients at the end of a treatment cycle with a minimum distance of four to six weeks to the next cycle (66, 75). However, these experientially suggested time points need further confirmation in clinical studies. Without doubt, the best strategy is to vaccinate patients before starting treatment with depleting therapies but this is not always feasible in clinical routine. Notably, the discussed studies focused mostly on humoral responses so that more information is needed on the role of the cellular immune response to immunization in patients under B cell-depleting therapies.
- High-dose corticosteroids are often employed for relapse treatment in MS. However, no studies on vaccine efficacy in this treatment regimen are available. Data about the impact of long-term low-dose glucocorticoid treatment on vaccine efficacy in patients with pulmonary or rheumatological diseases show small or no effects on humoral immune responses to non-live vaccines (48, 76, 77). Impaired immune responses after influenza vaccination were shown for patients with systemic lupus erythematodes receiving prednisone doses higher than 20 mg per day (78). Preliminary data on SARS-CoV-2 mRNA vaccination in patients with chronic inflammatory diseases showed reduced antibody titers and decreased neutralizing capacity when patients were receiving glucocorticoid therapy (71). In clinical practice, an interval of two to four weeks between immunization and high-dose corticosteroid MS relapse therapy is often recommended.
- In summary, most of the agents in use for the treatment of MS allow for the mounting of a protective immune response to vaccines but responses can be reduced under some of them. Timing may be a crucial factor for the purpose of enhancing vaccine efficacy.

## The Necessity of Measuring Virus-Specific Immune Responses in MS Patients After SARS-CoV-2 Vaccination

**Figure 1:**
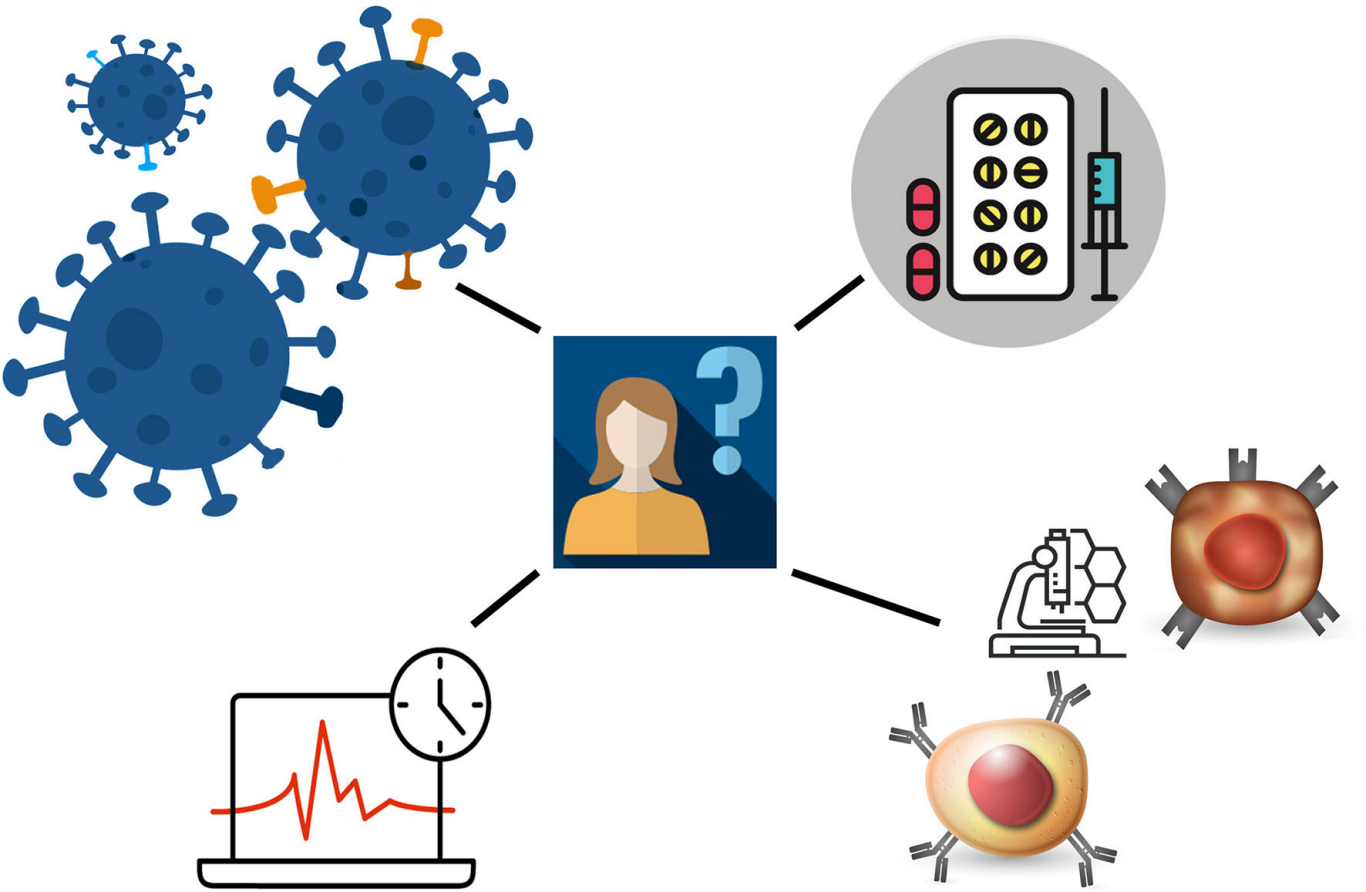
Open questions concerning SARS-CoV-2 vaccination in MS patients. Several issues regarding the vaccination against COVID-19 in MS patients still need to be addressed by further studies. For one, it is not clear, whether vaccination is as efficacious in patients receiving immunomodulating therapies as it is in healthy people. Second, the immunological equivalent of a protective immune response to vaccination has not been determined yet – are B cells and antibodies essential, is a specific T cell response needed? Furthermore, the durability of immune responses after vaccination is unknown. Lastly, it needs to be observed whether SARS-CoV-2 generates mutants that escape protection of a vaccine-elicited immune response.

We would like to encourage fellow physicians and scientists to measure both humoral and cellular immune responses to SARS-CoV-2 vaccination in MS patients for a number of reasons (**Figure 1**).

- First, the available evidence for vaccination under some of the DMT is still limited. For a few of them, only one or no published study at all is available (dimethyl fumarate, ozanimod, alemtuzumab, cladribine). Other therapies have only been investigated with regard to one type of vaccine (e.g., glatiramer acetate). Based on the data available until now, vaccination is most likely efficacious under treatment with beta-interferons, glatiramer acetate, teriflunomide, dimethyl fumarate, and natalizumab. By contrast, vaccine responses under S1P receptor modulators, B cell depletion, and high-dose corticosteroids are expected to be attenuated. Especially for the latter three types of immunomodulation, it is necessary to gather more evidence on the efficacy of immunization and the most suitable time point of vaccination in relation to the treatment cycle as the acquired data may have implications for future disease management and vaccination schedules.
- Second, all of the studies cited above were conducted with inactivated, polysaccharide, conjugate, toxoid vaccines, or neoantigens. However, currently authorized vaccines against SARS-CoV-2 in Europe and the United States are based on mRNA and viral vector technology. Vaccines from these platforms have never been examined in MS patients before so that not all of the available data concerning the efficacy of other vaccine types may be transferable (30).
- Third, we need to collect information on the durability of protective immune responses. As for the vaccine trials, immunological data concerning the adaptive humoral response are available for a maximum of 119 days after first vaccination with mRNA-1273 by Moderna (79–82), day 85 after the first administration of BNT162b2 by Pfizer-BioNTech (10, 83), day 71 after application of Ad26.COV2.S by Janssen (12, 84), and day 56 after the first vaccination with ChAdOx1 nCoV-19 by Oxford-AstraZeneca (11, 85, 86). Published data on the adaptive cellular response cover an even shorter time span after vaccination. After the aforementioned time points, we lack data on the further development of the adaptive immune response. Moreover, we do not know whether the data from the vaccine trials in the general population are also applicable to MS patients with and without DMT. Further research on this subject is urgently needed in order to be able to make sensible decisions about re-vaccination schemes in the future.
- Fourth, it will be necessary to monitor the evolution of SARS-CoV-2 mutations possibly escaping the vaccination-elicited immune response. There have been reports about circulating viral mutants that subvert the neutralizing capacity of antibodies elicited by natural infection or SARS-CoV-2 vaccination (87, 88). Less is known about the protective capacity of non-neutralizing SARS-CoV-2-specific antibodies. Data from animal models suggest that Fc receptor-mediated mechanisms might play a role in protection from SARS-CoV-2 infection as well (89). Possibly, such non-neutralizing antibodies are more robustly potent with regard to escape mutants as they usually target more conserved epitopes (87). Furthermore, the cellular response elicited by vaccination may be more diverse and cross-protective against escape mutants as already shown for influenza virus infection in mice (70, 90). It is necessary to pursue the protective capacity of vaccination against SARS-CoV-2 mutants in order to be able to react in a timely manner if vaccine adaptations turn out to be required. This is especially true for immunocompromised patients who might develop a less robust immune response after vaccination and might hence be more prone to infection with viral escape mutants.

## Protective Immune Response Against SARS-CoV-2

In most studies on vaccination efficacy in the past, humoral responses were measured as correlate for a protective immune response (48). Frequently used parameters include antigen-specific and neutralizing antibody titers or hemagglutination inhibition titers. However, measuring the humoral response alone is not always suitable for a precise characterization of virus-specific immune responses as already shown for the case of varicella-zoster virus (VZV) infection (91).

**Figure 2:**
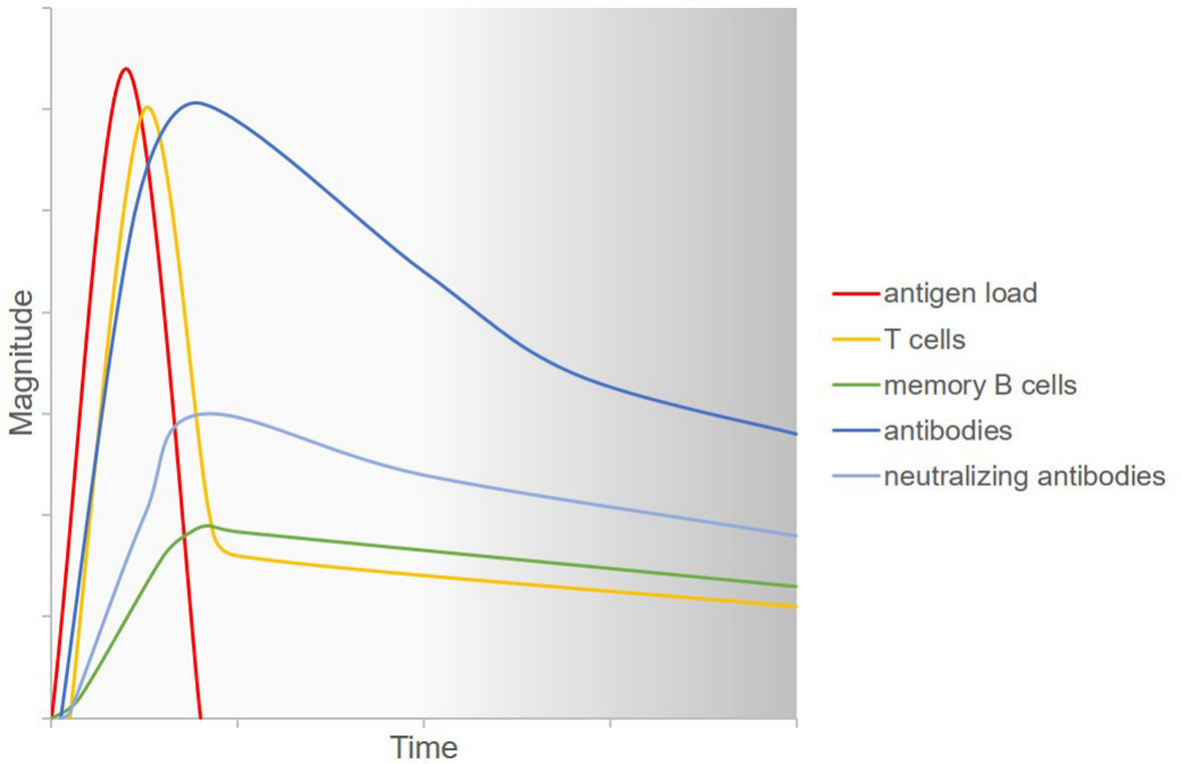
Current understanding of the adaptive immune response to SARS-CoV-2 infection. Upon infection with SARS-CoV-2, the virus replicates in the human host and hence antigen load increases. Virus-specific T cells are generated and fight the virus so that antigen load decreases. Simultaneously, B cells differentiate into plasma cells which produce antiviral antibodies. A part of the secreted antibodies have neutralizing capacities. Memory B cells emerge which can secrete specific antibodies more rapidly upon rechallenge with the antigen. All of the aforementioned agents of the immune system decrease over time. However, the long-term kinetics of this decrease and the response to a possible repeated viral challenge at various time points are not known. Figure adapted with permission from (92, 93).

The adaptive immune response after SARS-CoV-2 infection is characterized by heterogenous humoral and cellular responses which are furthermore interdependent (**Figure 2**). After COVID-19 disease, spike-binding and neutralizing IgG antibodies declined only slightly up until 8 months after infection (94). In asymptomatic individuals infected with SARS-CoV-2, however, titers of virus-specific and neutralizing antibodies started to decrease as soon as two to three months after infection (95). The number of virus-specific memory B cells increased until three months (96) and even up until eight months after infection (94). SARS-CoV-2-specific CD4+ and CD8+ T cells were shown to have a half-life of three to five months, even though a less pronounced decline can be expected beyond eight months after antigen exposition based on data from yellow fever virus, smallpox, and SARS-CoV (94). In another study, highly functional SARS-CoV-2-specific CD4+ and CD8+ T cells were detectable for more than three months after infection. Here, virus-specific memory CD4+ T cells exhibited T helper type 1 and type 17 cytokine profiles upon stimulation, hence producing the cytokines that are needed for class-switching to IgG and IgA (96). In asymptomatic and mild SARS-CoV-2 infection, the virus-specific T cell response was shown to be more pronounced than the antibody response and T cells exhibited antiviral functional properties even when antibodies were not detectable at all (6, 97).

The important question is which of these components of the adaptive immune system confers protection. It was shown that convalescent plasma transfer in COVID-19 patients was more efficacious when SARS-CoV-2-binding antibody titers in plasma were higher (98), leading to the assumption that virus-specific antibodies mediate protection against viral replication in infected patients and therewith might also protect from reinfection. Possibly, the amount of antibody itself is, however, not as relevant as the potential ability to produce antibody upon rechallenge. It was suggested that antigen-specific memory B cells may be more relevant than antibody levels for long-term immunity in some viral infection settings as the memory B cells are able to proliferate and differentiate into plasma cells upon reinfection (99–101). For this reactivation of memory B cells, interaction with memory CD4+ T cells is needed. In line with this, a correlation was shown between the CD4+ T cell response and SARS-CoV-2-specific IgG and IgA levels (6). Apart from providing help for B cells, virus-specific CD4+ and CD8+ T cells play a crucial role in protecting the organism from SARS-CoV-2 *via* other mechanisms (5–7, 9, 10). As a matter of fact, SARS-CoV-2-specific CD4+ and CD8+ T cells were each associated with milder disease in one study while neutralizing antibody titers were not (8). In patients with genetic agammaglobulinemia, T cells were able to protect from severe COVID-19 disease in the absence of B cells (102). For the closely related SARS-CoV, which was the cause for a global outbreak with high morbidity and mortality in 2002 and 2003 (103), it was shown that virus-specific memory CD8+ T cells may still confer protection at a time when antibodies and memory B cells are not detectable any more (104).

Immune correlates for protection against SARS-CoV-2 have not been defined yet. However, high titers of virus-binding and neutralizing antibody in combination with a CD4+ T helper type 1 response and a solid amount of SARS-CoV-2-specific cytotoxic CD8+ T cells seem to be suitable predictors of a reduced risk of infection with SARS-CoV-2 and of a decreased probability of severe COVID-19 disease (11, 12). The potential role of measuring antigen-specific memory B cells for the evaluation of long-term protective immunity should also be discussed for SARS-CoV-2 as has been proposed likewise for dengue virus, smallpox, and VZV (100, 101, 105, 106).

As discussed above, DMT modulate the immune system in MS patients and therewith the response to infectious agents and vaccination. It would be helpful to be able to discern the individual capacity to mount a protective immune response after vaccination based on immunological parameters. The most frequent laboratory finding in this context is lymphopenia, especially under therapy with S1P receptor modulators, B cell-depleting agents, and cladribine. SARS-CoV-2 itself often causes lymphopenia as well and the lack of lymphocytes can in turn lead to increased viral load and therewith disease severity. Lymphopenia even serves as a prognostic indicator of COVID-19 disease severity (107). It is imaginable that lymphopenia before SARS-CoV-2 infection as observed in MS patients under certain DMT hence also facilitates viral spread. First data by Achiron et al. on the immune response in MS patients after vaccination with BNT162b2 provided evidence that humoral vaccine responses were attenuated in most patients under treatment with ocrelizumab and fingolimod. For both therapies, the extent of the humoral response did not depend on the absolute lymphocyte count (108). Regarding ocrelizumab, the failure to produce vaccine-specific IgG antibodies is likely due to the depletion of the B cell lineage which is responsible for antibody production. As for fingolimod, the humoral vaccine response was also attenuated independently of the degree of lymphopenia. It was shown that the therapeutic effect of fingolimod is not linked to the absolute lymphocyte count, and it was suggested that it rather depends on the amount of CD19+ B cells and CD4+ T cells (109). Analogically, the failure to produce sufficient amounts of anti-SARS-CoV-2 antibodies may also be due to the lack of these specific subsets instead of the low absolute number of lymphocytes. Possibly, CD19+ B cell and CD4+ T cell counts may hence be a predictor of the efficacy of humoral vaccine responses in patients under DMT. Further studies will be needed to evaluate this hypothesis.

A less frequent laboratory abnormality in MS patients under DMT is hypogammaglobulinemia. It appears especially in patients who have been on B cell-depleting agents over a long period of time (30, 110). As mentioned above, B cell-depleted patients have an increased risk for contracting a SARS-CoV-2 infection and for severe COVID-19 disease. Patients with low antibody levels due to common variable immunodeficiency are more susceptible to severe COVID-19 disease as well. Thus, hypogammaglobulinemia may also be a predictor for higher susceptibility to and severity of COVID-19 in patients with acquired hypogammaglobulinemia (30, 111). Possibly, a reduced vaccine response can be expected in these patients as well. However, the impaired immune response is in all likelihood causally connected not to the low amount of pre-existing antibody levels but to the underlying lack of B cells.

Overall, there is an interdependence between the different immune compartments, but possibly also at least a partial redundance. The immunomodulation conferred by many DMT in MS patients does not impair the immune response as profoundly as nonselective immunosuppressive agents. B cell-depleted MS patients, for example, may profit from a robust virus-specific T cell response, even if antibodies are not produced sufficiently. This highlights the importance of examining both arms of the adaptive immune response.

## Measuring Humoral and Cellular Responses to Vaccination

We suggest measuring the humoral as well as cellular immune responses after SARS-CoV-2 vaccination in MS patients. Reasonable time points for the measurement of the adaptive immune response would be before the vaccination, and 4 and 8 weeks after administration of the first vaccine dose to evaluate the immunological efficacy of the vaccination. We propose recurrent measurements 6 and 12 months after the immunization in order to be able to estimate the durability of a protective immune response.

Certain caveats need to be addressed with respect to the analysis of vaccine responses. As for the analysis of anti-SARS-CoV-2 antibodies, multiple different assays are in use. On the one hand, there are assays for the detection of antibodies that bind different antigens of the virus, most frequently the spike protein or parts of it and the nucleocapsid. On the other hand, different assays are available evaluating the neutralizing properties of the antibodies including plaque reduction neutralization assays, microneutralization assays, and pseudovirus neutralization assays, amongst others. Partly, these tests have generated unreliable data and some of the available enzyme-linked immunosorbent assays (ELISA) for the measurement of binding antibodies have exhibited limited precision (112, 113). A new optimized quantitative test for SARS-CoV-2 spike- and nucleocapsid-binding IgG and IgM antibodies has recently been developed based on chemiluminescence enzyme immunoassay (CLEIA) technology with the goal of being able to determine a threshold for the definition of protective immunity soon (114).

The measurement of cellular responses poses certain problems as well. Frequently measured outcome parameters for the quantification of the amount and functionality of the T cell response include proliferation and cytokine secretion of T cells after stimulation with antigen peptide pools. Besides the composition and length of the peptides themselves, peptide concentration and number of peptides in the respective pool may significantly influence the results (115, 116).

Most studies addressing the immunogenicity of SARS-CoV-2 vaccines employed ELISA for the detection of spike protein- and receptor binding domain (RBD)-specific binding antibody, different forms of neutralization assays, interferon-gamma (IFN-µ) enzyme-linked immunospot (ELISpot) assays of peripheral blood mononuclear cells (PBMC), and intracellular cytokine staining of CD4+ and CD8+ T cells. SARS-CoV-2 spike protein- and RBD-binding antibody titers correlated tightly with neutralizing antibody titers in several vaccination studies (81, 84, 86, 117). In order to be able to compare our data with the data from the vaccine trials, we will measure SARS-CoV-2 RBD-specific binding antibody titers and conduct IFN-µ ELISpot assays of PBMC. Data acquired *via* a SARS-CoV-2 QuantiFERON^®^ test (QIAGEN^®^) measuring the IFN-µ secretion of lymphocytes stimulated with two SARS-CoV-2 antigen pools will be compared to the ELISpot data. By adding an ELISA for the detection of nucleocapsid-specific antibodies, we will gather information on whether patients may have been infected with SARS-CoV-2 before or after vaccination. The aforementioned will be conducted as an observational clinical study in our center. In parallel, we participate in prospective, multicenter, open-label, low-intervention phase IV clinical trials evaluating the use of SARS-CoV-2 mRNA vaccines in MS patients treated with siponimod or ofatumumab (NCT04792567 and EUDRACT 2021-000307-20, respectively).

## Conclusion

MS patients should be vaccinated against SARS-CoV-2 as soon as possible in order to reduce their infection and associated relapse risk. Extrapolating from other studies with non-live vaccines, application of currently authorized SARS-CoV-2 vaccines is expected to be safe in patients with MS. However, vaccination may induce an attenuated immune response in patients under certain DMT and evidence on sensible time points for vaccination, especially in B cell-depleting therapy, is currently lacking. Therefore, we propose to closely survey MS patients after vaccination and to measure their humoral and cellular responses to SARS-CoV-2 vaccination.

## Data Availability Statement

The original contributions presented in the study are included in the article/supplementary material. Further inquiries can be directed to the corresponding author.

## Author Contributions

CW wrote the manuscript and created the figures. TZ designed and revised the manuscript. KA and KS provided critical feedback and are in charge of conducting the described studies in our center. All authors contributed to the article and approved the submitted version.

## Conflict of Interest

TZ received personal compensation from Biogen Idec, Bayer, Novartis, Sanofi, Teva, and Synthon for consulting services. TZ received additional financial support for research activities from Bayer, Biogen Idec, Novartis, Teva, and Sanofi Aventis. TZ is principal investigator of the AMA-VAC and KYRIOS study. KA received personal compensation from Roche, Novartis, Sanofi, and Celgene for consulting services.

The remaining authors declare that the research was conducted in the absence of any commercial or financial relationships that could be construed as a potential conflict of interest.
